# Editorial: Regulation of photosynthesis: redox dynamics and environmental adaptations

**DOI:** 10.3389/fpls.2026.1887488

**Published:** 2026-06-18

**Authors:** Thomas Roach, Linda de Bont, Haijun Liu

**Affiliations:** 1 Department of Botany, University of Innsbruck, Innsbruck, Austria; 2 Université de Lorraine, INRAE, IAM, Nancy, France; 3 Department of Biology, Saint Louis University, St. Louis, MO, United States

**Keywords:** chlorophyll, photoprotection, photoregulation, photosynthesis, proton motif force, redox regulation

This Research Topic explores the fundamental mechanisms underlying light-driven redox reactions in photosynthetic organisms—a process critical to life on Earth. Oxygenic photosynthesis has generated the vast majority of organic carbon and oxygen in our atmosphere, effectively powering nearly every food chain on the planet.

At its core, and specific to this topic, photosynthetic electron flow serves a dual purpose: it enables NADPH production and drives electron-coupled proton transport that powers ATP synthesis, which collectively must achieve the correct NADP: ATP availability to meet shifting metabolic demands. Because the ratios fluctuate according to environmental conditions, organisms have evolved a sophisticated suite of regulatory mechanism.

The four studies featured in this Research Topic seek to answer questions related to the regulation of photosynthesis at molecular and cellular levels in response to environmental changes. Their collective goal is to provide insights that could bypass the current 6% photosynthetic efficiency limit in most crops. These works highlight how proton motive forces, bicarbonate chemistry, redox-sensitive enzyme regulation, and CO_2_-responsive respiration collectively shape photoprotection, energy efficiency, and carbon gain and, importantly, by doing so expose the limits of our current models and measurement approaches.

## 1 The proton motive force as a central regulator

In their review *“Proton motive force partitioning links energy and redox balance to photoprotection and carbon gain”*, Didaran et al., describe in detail how the proton motive force (pmf) acts as a central regulator of photosynthetic performance and photoprotection. The thylakoid pmf is partitioned energy currency comprised of a chemical gradient (ΔpH) and an electrostatic component (Δψ). The balance between these two facets, and the kinetics by which they are established or relaxed, determine how quickly photosystems respond to light fluctuations and how efficiently energy is allocated to synthesis of ATP and metabolism. The rapid formation of ΔpH during increases in light intensity enables activation of high-energy state quenching type non-photochemical quenching (qE-type NPQ) via photosystem II (PSII) subunit S (PsbS), and photosynthetic control at cytochrome *b*_6_*f* (Cyt *b*_6_*f*). Counter-current ion transporters, such as VCCN1 and CLCe that promote rapid ΔpH formation, and KEA3 that accelerates ΔpH relaxation after transitions to lower light, are described as molecular regulators which plants use to tune photoprotection and energy throughput on second-to-minute timescales. They highlight what knowledge remains inferred versus what can be directly measured. Finally, a multi-measurement and non-destructive set of techniques, comprising multi-wavelength electro-chromic shift (ECS) measurements (to quantify pmf), chlorophyll fluorescence quenching (to quantify NPQ) near-infrared absorbance measurements (to quantify P700/PSI redox kinetics), coupled to gas exchange, is proposed to help unravel the interaction of each regulatory component under changing environments, aiding attempts to improve photosynthetic efficiency in the field.

## 2 Bicarbonate: more than just a photosystem II cofactor

In *“Depletion reveals role of bicarbonate in the photosynthetic electron transport chain of Limnospira maxima”*, Castillo et al., investigate the crucial role of bicarbonate in regulating proton-coupled electron transfer and energy distribution within the photosynthetic apparatus of this cyanobacteria. It is well known that bicarbonate binds to the non-heme iron between the primary and secondary PSII electron acceptors, Q_A_ and Q_B_, and that its depletion perturbs PSII activity. Here, it is shown the donor and acceptor sides of PSII are impaired while destabilizing phycobilisome connectivity, thus that bicarbonate is a multifaceted regulator of photosynthesis. Data showed that under bicarbonate depletion, some PSII centres retained water-oxidizing capacity, but that electron flux was constrained. Specifically, after depletion, the transport of more than two electrons from Q_A_ to Q_B_ was not possible. Beyond PSII, bicarbonate depletion altered Cyt *b*_6_*f* reoxidation kinetics and delayed P700 reoxidation kinetics, suggesting impaired carbon fixation and donor-side limitation at PSI. These broader effects suggest that bicarbonate is critical beyond maintaining PSII electron flow and highlights future directions and considerations for investigation of other known PSII bicarbonate depletion techniques.

## 3 Redox control of pigment biosynthesis

In *“Redox regulation of glutamate-1-semialdehyde aminotransferase modulates the synthesis of 5-aminolevulinic acid in Arabidopsis”*, Sinha et al., investigate metabolic side of regulation of a key tetrapyrrole (and therefore chlorophyll) biosynthesis step. The identification of Cys168 and Cys190 as crucial redox-sensitive residues in glutamate-1-semialdehyde aminotransferase (GSAAT) reveals a post-translational mechanism by which the cellular redox environment can tune the flux into the 5-aminolevulinic acid (ALA) node, the rate-limiting step in the synthesis of chlorophyll and heme. Oxidation-induced disulfide formation in GSAAT reduces its activity, while reduction of the disulfide increases it, shedding light on how plants might modulate pigment biosynthesis in response to environmental cues that perturb redox homeostasis. This redox switch adds a new dimension to energy-redox-carbon balancing. In fluctuating light or stress, such regulation could increase the plant’s capacity to assemble and replenish pigment pools, with downstream consequences for light harvesting, photoprotection, and photosynthetic resilience.

## 4 Refining models of respiration and carbon flux

In the study *“Accuracy of photorespiration and mitochondrial respiration in the light fitted by CO_2_ response model for photosynthesis”*, Niu et al., evaluate how photorespiration (R_p_) and mitochondrial respiration (R_d_) respond to varying CO_2_ concentrations by compared to modelled values. Their findings challenge several widely held paradigms in carbon flux modelling. They find that *A*/C_a_ values provide superior fit to measured respiration, as compared with *A*/C_i_, indicating that atmospheric rather than intercellular CO_2_ concentrations are more relevant. Furthermore, disparities between model-derived R_p_ and R_d_ and measured values reveal limitation in current modelling. The observed, dose-dependent, non-linear relationship between ambient CO_2_ and R_p_ or R_d_, with distinct species thresholds for peak respiration and photorespiration, challenges linear assumptions and highlights interspecific variation. These results imply that climate-change-relevant predictions of crop carbon gain cannot rely on generic, linearized CO_2_-respiration relationships. The authors conclude that future models must integrate CO_2_-responsive respiratory control with photosynthetic regulation, accounting for the dynamic interplay between chloroplasts and mitochondria under non-steady-state.

